# Factors Influencing the Yield of Progenitor Cells in Bone Marrow Aspiration Concentrate—A Retrospective Analysis of 58 Patients

**DOI:** 10.3390/biomedicines11030738

**Published:** 2023-03-01

**Authors:** Sathish Muthu, Madhan Jeyaraman, Aditya Narula, V. R. Ravi, Avinash Gandi, Manish Khanna, Nicola Maffulli, Ashim Gupta

**Affiliations:** 1 Indian Stem Cell Study Group (ISCSG) Association, Lucknow 226010, Uttar Pradesh, India; 2 Department of Orthopaedics, Government Medical College and Hospital, Dindigul 624003, Tamil Nadu, India; 3 Department of Biotechnology, School of Engineering and Technology, Sharda University, Greater Noida 201310, Uttar Pradesh, India; 4 Orthopaedic Research Group, Coimbatore 641045, Tamil Nadu, India; 5 Department of Orthopaedics, ACS Medical College and Hospital, Dr. M.G.R. Educational and Research Institute, Chennai 600056, Tamil Nadu, India; 6 Department of Regenerative Medicine, Mother Cell Regenerative Centre (MCRC), Tiruchirappalli 620017, Tamil Nadu, India; 7 South Texas Orthopaedic Research Institute (STORI Inc.), Laredo, TX 78045, USA; 8 Department of Orthopaedics, Aakaar Bone Care, Kanpur 208002, Uttar Pradesh, India; 9 Department of Musculoskeletal Disorders, School of Medicine and Surgery, University of Salerno, 84084 Fisciano, Italy; 10 San Giovanni di Dio e Ruggi D’Aragona Hospital “Clinica Ortopedica” Department, Hospital of Salerno, 84124 Salerno, Italy; 11 Barts and the London School of Medicine and Dentistry, Centre for Sports and Exercise Medicine, Queen Mary University of London, London E1 4DG, UK; 12 School of Pharmacy and Bioengineering, Keele University School of Medicine, Stoke on Trent ST5 5BG, UK; 13 Future Biologics, Lawrenceville, GA 30043, USA; 14 BioIntegrate, Lawrenceville, GA 30043, USA; 15 Regenerative Orthopaedics, Noida 201301, Uttar Pradesh, India

**Keywords:** BMAC, bone marrow aspiration concentrate, cellular yield, progenitor cells, regenerative medicine, mononuclear cell, colony-forming unit

## Abstract

This study aims to identify the role of subjective factors (age, sex, and comorbidities) and procedure-specific factors (aspiration volume) in influencing the yield of progenitor cells in bone marrow aspiration concentrate (BMAC) harvested from the iliac crest. A retrospective analysis was conducted on 58 patients (male:female = 31:27; mean age: 52.56 ± 18.14 years) who underwent BMAC therapy between January 2020 and June 2021. The factors analyzed include individual factors such as age, sex, and comorbid conditions, and procedural factors such as aspirate volume. The mononuclear cell (MNC) count and colony-forming unit (CFU) assay were used to assess the yield of progenitors in the aspirate. Pearson’s correlation test was performed for the age, aspirate volume, and outcome parameters, such as MNC and CFU. We used the chi-square test to analyze the role of sex and comorbidities on cellular yield. The mean volume of aspirate used for BMAC therapy was 66.65 (±17.82) mL. The mean MNC count of the BMAC was 19.94 (±16.34) × 10^6^ cells, which formed 11 (±12) CFUs. Evidence of statistically significant positive associations was noted between the CFUs developed from the BMAC and the MNC count within them (r = 0.95, *p* < 0.001). The sex of the individual did not play any significant role in MNC count (*p* = 0.092) or CFUs formed (*p* = 0.448). The age of the individual showed evidence of a statistically significant negative association with the MNC count (r = −0.681, *p* < 0.001) and CFUs (r = −0.693, *p* < 0.001), as did the aspiration volume with the MNC count (r = −0.740, *p* < 0.001) and CFUs (r = −0.629, *p* < 0.001). We also noted a significant reduction in the MNC count (*p* = 0.002) and CFUs formed (*p* = 0.004) when the patients presented comorbidities. Individual factors such as age, comorbid conditions, and procedure factors such as aspirate volume significantly affected the yield of progenitor cells in the BMAC. The sex of the individual did not influence the yield of progenitor cells in BMAC.

## 1. Introduction

With the revolution in molecular biology, personalized medicine and customization have gained global popularity among healthcare providers [1]. Bone marrow aspirate concentrate (BMAC) has been widely used to regenerate musculoskeletal tissues [2,3]. BMAC is a heterogeneous group of cells comprising bone marrow-derived mesenchymal stromal cells (BM-MSCs), hematopoietic progenitor cells, fibroblasts, macrophages, and endothelial precursor cells [4,5]. In the bone marrow, the concentration of BM-MSCs contributes to 0.001% to 0.01% of cells [6,7]. BM-MSCs have greater osteogenic than chondrogenic and adipogenic potential [8,9,10,11]. The plasticity of MSCs is species-dependent [12]. To target regeneration of musculoskeletal tissues, bone marrow is concentrated to provide an adequate number of BM-MSCs [13,14,15]. 

There are variable and discrepant results with the use of BMAC in various clinical settings [16,17,18,19,20,21]. The discrepancies in the cellular yield of BMAC may arise from differences in the age and sex of the individual, the presence of co-morbidities, the source of bone marrow, and the method of isolation and characterization [22,23]. The various factors influencing the cellular yield of BMAC include sources of BM-MSCs (autologous or allogenic), choice of anesthesia, site of bone marrow aspiration, type of syringe used for aspiration, anticoagulant of choice, type of bone marrow aspiration needle used, speed and time of centrifugation, and volume of the final injectate to be delivered at the target site [24]. Apart from these process variables, individual patients’ subjective factors such as age, sex, and co-morbidities (diabetes mellitus, hypertension, rheumatoid arthritis, and any systemic illness) play a major role in the cellular yield of BMAC [25]. Various studies have investigated the role of aging in the yield of BM-MSCs [26,27,28]. Dragoo et al. observed variability in the cellular mixture of BMAC prepared using various commercially available kits (Harvest SmartPrep 3, Biomet BioCUE, and Arthrex Angel) [5]. Recent studies have analyzed the role of age in the yield of BM-MSCs from BMAC [29], but the role of comorbidities and procedure variables is still unclear. The present study analyzed the influence of subjective factors such as age, sex, comorbidities, and procedure-specific variables, such as aspiration volume, on the cellular yield of progenitor cells in BMAC harvested from the iliac crest. 

## 2. Materials and Methods

This study was approved by the Institutional Ethical Committee from KSR Institute of Dental Sciences and Research (protocol code 127/KSRIDSR/EC/2016 and 25.04.2016), and informed consent was obtained from all subjects involved in the study. We performed a retrospective analysis of prospectively collected data from the records of patients undergoing bone marrow aspiration concentrate therapy for any etiology. We excluded patients without data regarding the cellular yield of their bone marrow aspirates. We extracted the anonymized patient data from the hospital records, including age, sex, comorbidities, the condition for which BMAC was administered, and process variables such as aspiration volume. 

The marrow was collected by a single experienced orthopedic surgeon from the anterior superior iliac crest of each patient. The samples were brought to the cell processing laboratory and were immediately processed. The bone marrow was processed to isolate mesenchymal stem cells as previously described by Pösel et al., 2012, with some modifications. Briefly, the aspirated bone marrow was diluted with plain medium (MesenPRO RS™, Gibco^®^ Life Technologies™, Grand Island, NY, USA) at a ratio of 5:2. The mixture was rinsed well and sieved through a 100 mm cell strainer (Gibco, Life Tech, Grand Island, NY, USA) to dissolve the remaining cell aggregates. We used hypotonic ammonium chloride buffer (Himedia R075, India) to lyse the RBCs present in the solution through short-term incubation for 30 s. This solution was layered over HiSep 1.077 (Himedia R075, India) to isolate human mononuclear cells, and then centrifuged at 400× *g* for 40 min. The bone marrow mononuclear cell (BMNC) buffy coat layer was then collected and washed in the plain medium. The final aspirate was reconstituted in 2 mL of the buffer medium. 

An appropriate volume of final aspirate was mixed with 0.4% Trypan blue of equal volume and mixed gently. The cells were maintained at room temperature for 5 min. Then, 10 µL of cells from this staining process were processed through a hemocytometer to estimate the number of unstained viable cells and stained dead cells. The average number of unstained cells in each quadrant multiplied by 2 × 10^4^ gives the total number of viable cells per mL. The number of viable cells divided by the number of dead and viable cells gives the percentage of viable cells. The MNC counts were expressed as 10^6^ cells per ml of the cell suspension. 

**Figure 1:**
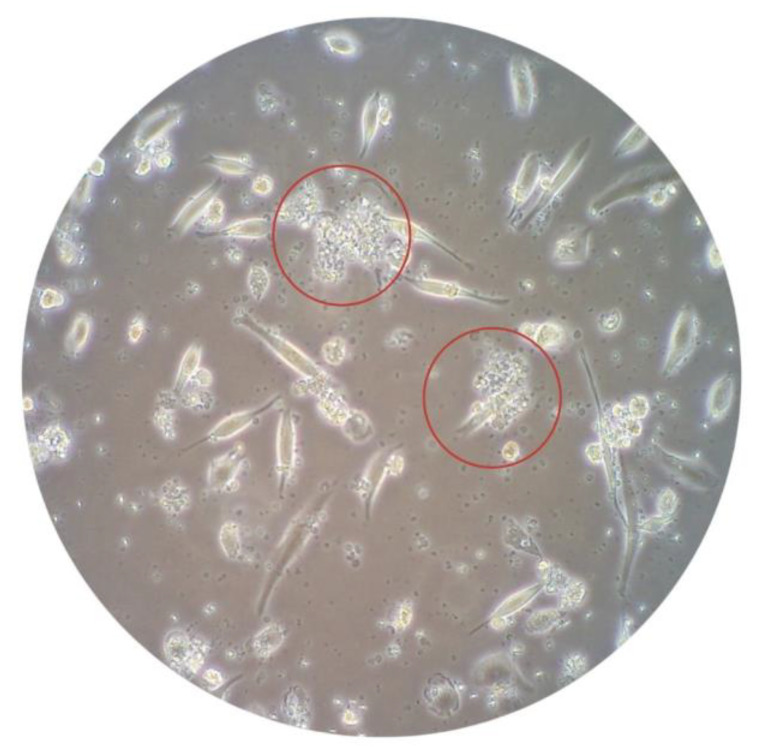
Microscopic appearance of the colonies in the CFU assay (200×). Red circles mark the identified colonies at 7 day culture.

The cell pellets from the BMAC were seeded to t25 tissue culture flasks containing MesenPRO RS™ Complete medium. The medium consisted of the basal medium supplemented with 20% growth supplement (Gibco^®^ Life Technologies™, NY, USA), L-Glutamine, to a final concentration of 2 mM, 100 U/mL penicillin, 100 μg/mL streptomycin, and 25 μg/mL amphotericin B (Gibco^®^ 15,240, Life Technologies™, NY, USA). The final count of CFU was estimated per 25 cm^2^ after a culture period of 7 days upon attaining cellular confluence. We considered a cell cluster of a minimum of 50 cells at 7 days of culture to be a colony, as illustrated in Figure 1. 

**Figure 2:**
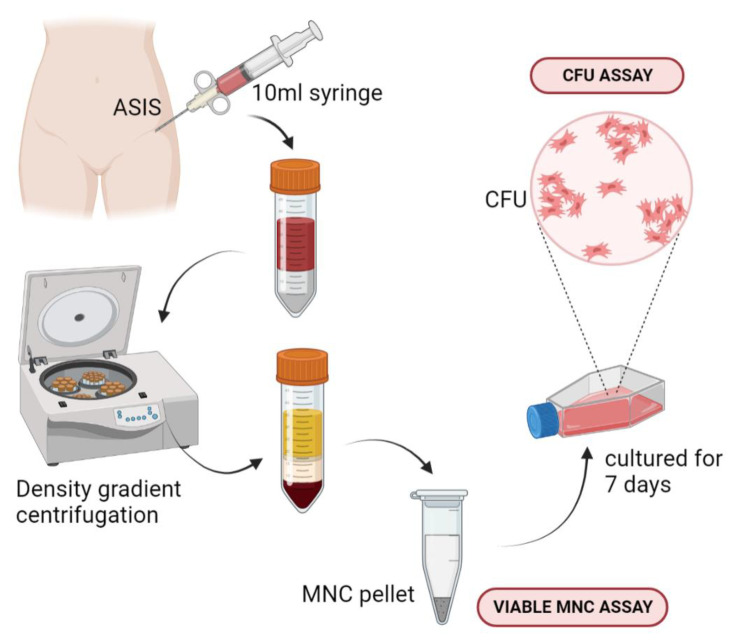
Schematic representation of bone marrow aspirate concentrate isolation and culture. ASIS: anterior superior iliac spine, MNC: mononuclear cell, CFU: colony-forming unit.

We noted variability in the volume of bone marrow aspirates used in the preparation of the BMAC based on the treated pathology. To standardize the values of MNC and CFU, which directly depend on the volume of bone marrow aspirate that was used in the preparation of the BMAC, we re-calculated the MNC and CFU counts per 10 mL of the bone marrow aspirate. The entire procedure is illustrated in Figure 2.

The statistical analysis was performed in IBM SPSS Version 25 (Chicago, IL, USA). We used the Shapiro–Wilk test to test the normality of the data. We used medians and interquartile ranges (IQRs) to present the continuous variables and percentages to present the categorical variables. We analyzed the association between the age, aspiration volume, and outcome parameters with Pearson’s correlation test. We made a cut-off to estimate the effect of the subjective categorical variables, such as sex and comorbidities, on the MNC count and CFU assay values using ROC analysis. We analyzed the significance of their association with the outcome parameters using the chi-square test and their degree of significance, which was represented in the odds ratio. A *p* value lower than 0.05 was considered statistically significant. 

## 3. Results

A total of 58 patients (median age: 60 (40–66) years; 31 male and 27 female patients) underwent BMAC therapy (Table 1). Of the 58 patients, 9 (15.5%) had diabetes mellitus and 4 (6.8%) had rheumatoid disorder; the remaining 45 (77.5%) patients had no comorbidities. BMAC was employed in the management of a variety of conditions, the most predominant of which were osteoarthritis (n = 32), diabetic foot ulcers (n = 8), and rheumatoid arthritis (n = 4). The median volume of bone marrow aspirated was 70 (50–80) mL. The median MNC count and CFU were 16.27 (11.17–20.66) × 10^6^ cells and 7.33 (5–12.33) colonies for every 10 mL of bone marrow aspirate, respectively. 

### 3.1. Age

**Figure 3:**
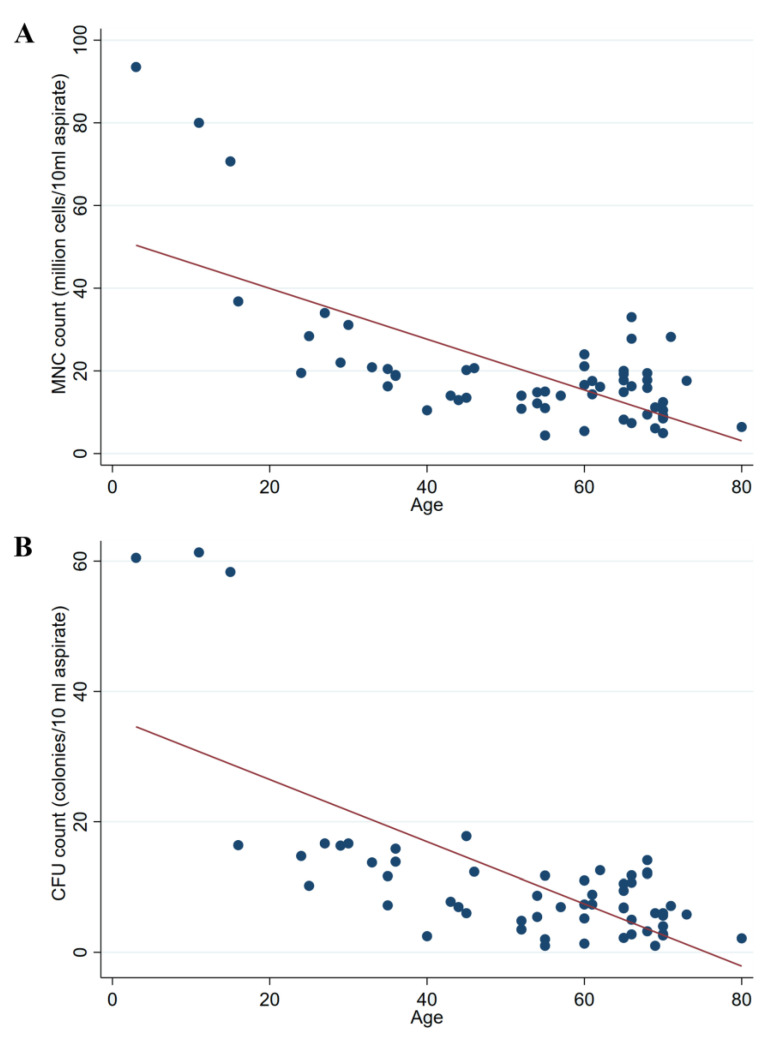
(**A**) Association scatter plot of age of the patient and MNC count of the formed BMAC. (**B**) Association scatter plot of age of the patient and formed CFU count.

There was evidence of a statistically significant negative association between the age of the patient and the MNC count (r = −0.681, *p* < 0.001) and CFUs (r = −0.693, *p* < 0.001) (Figure 3). 

### 3.2. Sex and Comorbidities

**Figure 4:**
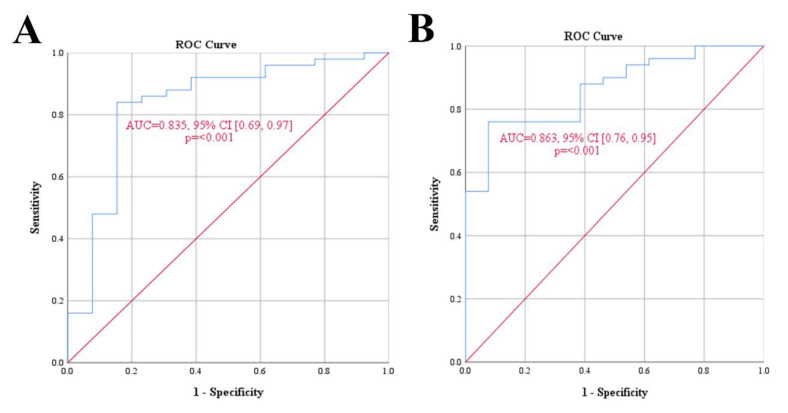
ROC curve estimation of the cut-off to analyze the relationship of the MNC count and CFU with categorical variables such as comorbidities. (**A**) ROC curve for MNC count and comorbid illness. (**B**) ROC curve for CFU count and comorbid illness.

We first identified the critical cut-off to evaluate the relationship between the categorical variables, such as sex and comorbidities, with the outcome variables, namely the MNC and CFU counts. For the MNC counts, based on the ROC analysis, 10 million cells was identified as the cut-off, with an area under the curve of 0.835, CI = 0.69 to 0.97, and *p* =< 0.001 (Figure 4A). Similarly, for the CFU assay, 3 colonies was identified as the cut-off, with an area under the curve of 0.863, CI = 0.76 to 0.95, and *p* =< 0.001 (Figure 4B). There were no significant differences concerning sex among the included patients regarding their MNC count (*p* = 0.092) or their ability to form CFUs (*p* = 0.448). 

The presence of comorbidities significantly reduced the cellular yield from the aspirate (*p* = 0.003). Patients without comorbidities had 9.85 times (CI = 2.21, 43.92) greater odds of a significantly high cellular yield (≥10 million cells) than those with comorbidities. Similarly, comorbidities significantly reduced (*p* = 0.005) the odds of the BMAC forming ≥3 regenerative CFUs by 14.7 times (CI = 1.38, 156.18) compared to those without any comorbidities (Table 2 and Table 3, respectively). Further exploring the impact of the individual comorbidities, diabetes significantly reduced the MNC count (OR = 9.2, 95% CI [1.740–48.634], *p* = 0.009) and CFUs formed (OR = 9.2, 95% CI [1.740–48.634], *p* = 0.0076). Similarly, rheumatoid arthritis significantly reduced the MNC count (OR = 11.5, 95% CI [1.261–104.864], *p* = 0.030) and CFUs formed (OR = 27, 95% CI [2.3–311.184], *p* = 0.0082). 

### 3.3. Aspiration Volume

**Figure 5:**
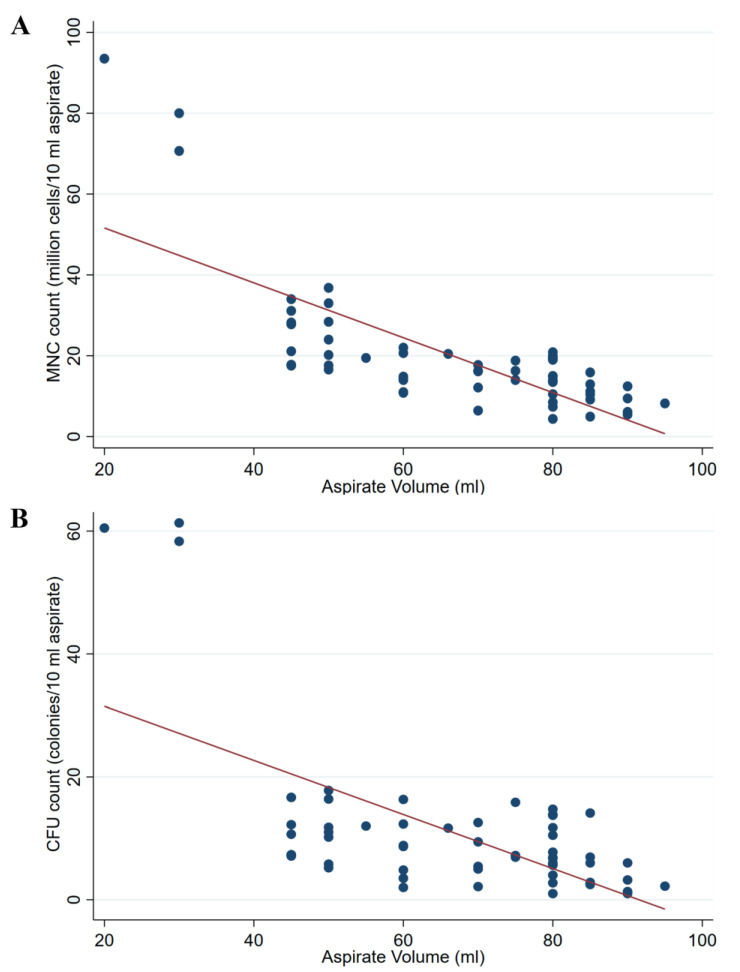
(**A**) Association scatter plot of aspiration volume of bone marrow and MNC count of the formed BMAC. (**B**) Association scatter plot of aspiration volume of bone marrow and formed CFU count.

Analyzing the impact of the aspiration volume on the MNC count and CFU count from the included patients, the aspiration volume showed evidence of a statistically significant negative association with MNC count (r = −0.74, *p* < 0.001) and CFUs (r = −0.629, *p* < 0.001) (Figure 5A,B, respectively). Thus, an increased volume of the aspirate did not guarantee an increased MNC count or CFU count, and most of the volume was accounted for by sinusoidal blood dilution into the aspirate from the bone marrow when aiming for high volume aspiration. The overall correlations between the factors analyzed in this study are presented as an association heat map (Table 4).

## 4. Discussion

Various sources, including adipose tissue, the umbilical cord, Wharton’s jelly, placenta, amniotic fluid, dental pulp, and hair follicles, can be used to isolate MSCs, but bone marrow remains a very versatile source for orthopedic surgeons [30]. In bone marrow, however, the absolute MSC count remains low; this is a limiting factor in applying MSC-based therapy in a clinical setting [31]. Theoretically, bone marrow aspiration needles with multiple holes improve the efficacy of the cellular yield of BMAC from bone marrow. Tanikawa et al. reported no significant differences between MNC counts derived from bone marrow needles, either with side holes or without side holes [32]. Apart from the iliac crest aspirates, the residue of reamer irritator aspirate was also shown to possess similar proliferative potential [33]. 

There is an age-related reduction in the cellular yield and adipogenic potential of MSCs compared to their osteogenic and chondrogenic potential [27]. In vitro, the MSC population, doubling rate, colony-forming units, and differentiation ability decline in BMAC samples procured from individuals of older age groups when compared to those from younger populations [26,34]. Thus, aging exerts a negative impact on the differentiation potential of BM-MSCs. Bellantuono et al. described age-related changes in the MSC pool, proliferation and differentiation capacity, and bone microarchitecture in BMAC concentrate [35]. Our results agree with the recent results by Cavallo et al. [29] concerning the age and yield of BMAC.

A statistically significant negative association was observed between the number of CFUs and aging in terms of the prevalence of alkaline phosphatase-positive CFUs; this occurs more rapidly in younger age groups, whilst older patients demonstrated a reduced rate of decline [36]. The MSCs derived from populations above 60 years show accelerated senescence compared to younger individuals, probably a consequence of the upregulation of gene expression for apoptosis and cellular senescence of MSCs in older subjects. Zhou et al. reported 4-fold upregulated cellular senescence of MSCs in donors older than 55 years [26]. Stenderup et al. and Justesen et al. reported no significant difference in the number of MSC colonies derived from healthy human donors aged 66 to 78 years [37,38]. The BMAC cellular fractions of leukemia patients show lower yields than those of healthy individuals, but no histomorphological changes were observed between leukemia patients and normal individuals [31,39,40]. However, Ganguly et al. demonstrated a decline in the number of MSCs per mL of bone marrow aspirate with age [41]. Similarly, the MSCs of children have been shown to exhibit lower doubling time upon culture and higher CFU counts [42]. The results of our study, based on CFU assay (r = −0.69, *p* < 0.001), validated the findings of this study (r = −0.52, *p* < 0.0001). Further, the researchers did not note any significant difference in the gene expression profile of these MSCs with age, thereby validating the use of BM-MSCs from elderly patients for autologous regenerative applications.

Betelsky et al. [43] studied the age and sex differences in the cellular yield of BMAC in 36 healthy patients without comorbidities. They observed a significant difference between young (3.075 MSCs per μL) and old (1.57 MSCs per μL) patients in terms of their MSC yield (*p* < 0.001). The cellular fraction in the BMAC of males (12.70 MSCs per μL) and females (17.39 MSCs per μL) was statistically significantly different (*p* < 0.001); increasing age and female sex significantly reduced the cellular fraction yield in BMAC preparation. However, Cavallo et al. [29] recently clearly showed that sex does not play a role in the yield of BMAC. This is in concordance with the results of the present investigation. 

Sexual dimorphism plays a major role in the central immune functions of MSCs. Dendritic cells in women produce more IFN-α and, hence, possess greater biological activity counts of neutrophils and macrophages than men. Pro-inflammatory surges, such as IL-6, TNF-α, and NK cell activity, are enhanced in men, whereas cellular and humoral responses are stronger in women [44,45]. In MSCs, 35 of 40 genes were attributed to sex-based differences. The effect of sex as a variable on the cellular yield of progenitors in BMAC and their inter-communication signaling pathways are not fully understood. More studies should be performed to analyze sex-based differences between the donor and the recipient to help clinicians to better understand cross-sex cellular transplantation [25]. 

No data are available on the effect of systemic diseases on the cellular yield of the progenitors in BMAC extraction. However, in the present study, the samples from patients with diabetes mellitus and rheumatoid arthritis demonstrated lower cellular yields of BMAC than the samples from patients without comorbidities. Further investigations should be planned to analyze the effect of other common comorbidities on the cellular yield of BMAC.

Li et al. reported a lower yield of BMAC injectate after repeated aspirations (a 66% and 74% decrease, from 87 per million MNC in the 1st aspiration to 30 per million MNC in the 10th aspiration, and to 22 per million MNC in the 20th aspiration, respectively) from the same site; this may include results from either peripheral blood dilution or decreased MSC count that are out of proportion to the decreased MNC counts [31]. This is in accordance with the results of the present study. The MSC colonies of males and females consisted of 107 and 46 units per million MNC, respectively (*p* = 0.12). There was no evidence of a statistically significant association between sex and the yield of MNC counts in either Pearson’s or Spearman’s correlation [31].

To obviate all of the aforementioned subjective factors affecting the yield of the cellular mixture of BMAC, allogenic BM-MSCs have been developed for various indications and are under analysis for approval for common clinical conditions. Jeyaraman et al. reported that the autologous source is superior to allogenic sources of MSCs in terms of safety, efficacy, and functional outcome of knee osteoarthritis [46]. However, the Drugs Controller General of India (DCGI) has recently approved allogeneic BM-MSCs for two indications, namely (a) critical limb ischemia (REGENACIP—200/100 million allogeneic cultured, pooled BM-MSCs), and (b) knee osteoarthritis (STEMONE—25 million allogeneic cultured, pooled BM-MSCs) after safety and efficacy studies. Allogenic MSCs do not impose any donor site morbidity, but there are still concerns regarding their immunogenicity. Moreover, allogenic MSCs allow donor selection and off-the-shelf utilization without the need for staged procedures, as is necessary for expanded autologous BM-MSCs. Repeated exposure to allogeneic MSCs may result in an adaptive immune response [47]. Large high-quality randomized controlled trials are needed to prove the safety, clinical efficacy, and cost-effectiveness of allogenic MSCs. 

Apart from the mesenchymal stromal cells, the mono-nuclear cell component of the BMAC constitutes osteogenic progenitors such as osteoblasts, fibroblasts, endothelial progenitor cells, pericytes, lymphocytes, and macrophages [13,14]. These cells play important roles in the regenerative cascade through angiogenesis, immunomodulation, etc. [15,48]. However, the impact of the individual components on the therapeutic efficiency of the BMAC needs further exploration [49].

**Figure 6:**
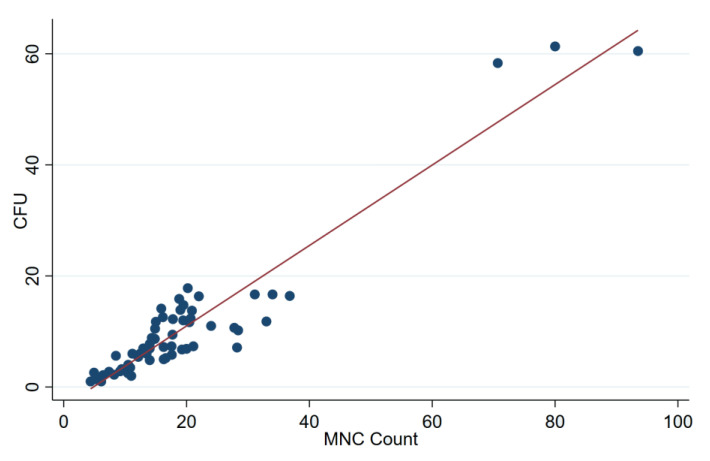
Correlation scatter plot of the MNC count of the BMAC and the CFUs formed from them.

The management of osteoarthritis is becoming more patient-specific with the development of phenotypes, molecular endotypes, and theratypes to tailor and optimize treatment [50]. The subjective factors identified in the present study would also play a role in optimizing the available biological treatment approaches. Following the identification of factors responsible for the yield of progenitor cells in the bone marrow aspirate, the next step in standardizing the process lies in the quantification of the composition of the MSC subpopulations and growth factors using cellular markers [50]. With these standardization techniques, consistency of the results could be ensured. Apart from the standardization of cellular components in the BMAC, standardization of the preparation process also needs to be ensured for optimal results [24]. The spectrum of indications for BMAC therapy is expanding, and presently already includes osteogenesis, neurogenesis, and the management of osteoarthritis, spinal cord injury, and femoral head osteonecrosis [51]. The CFU assay provides a simplistic measure of the stemness of the MSC in the BMAC [52]. A statistically significant positive association was noted between the CFUs developed from the BMAC and their MNC counts (r = 0.95, *p* < 0.001) (Figure 6). Hence, MNC count could be used as a surrogate marker of the ability of the MSCs within them to effectively form CFUs. Although we noted a strong positive correlation of the MNC count in the BMAC with the potential of the MSCs within them to form CFUs, the clinical implications of this correlation need further validation. 

The study has some limitations. First, although the data were collected prospectively, the analysis was undertaken retrospectively. Second, we were not able to analyze all the patients’ factors, such as obesity and procedure-related variables, that would have altered the yield of the BMAC obtained. Third, the proportion of patients with comorbidities was small, thereby precluding a one-to-one age-matched comparison. Moreover, we also did not analyze the effects of medications that these patients were taking for the management of their primary illness or comorbidities. Nevertheless, within these constraints, the obtained results are medically and biologically plausible, as well as scientifically valid. 

The current study has identified the roles of patient-related factors, such as age, sex, and comorbid conditions, as well as procedure factors, such as aspirate volume on the yield of MSCs in BMAC. However, apart from the analyzed factors, other patient-related factors that modify the stemness of MSCs include the use of glucocorticoids, alcohol, blood cell disorders such as sickle cell anemia, Gaucher disease, autoimmune conditions, diabetes, and chronic kidney diseases [52]. Further analysis into the effect of these confounders on the overall biology of the derived MSCs is needed. 

BMAC appears to a viable source of MSCs under the umbrella of minimally manipulated autologous products for use in various autologous applications, including inflammatory and degenerative disorders such as femoral head osteonecrosis [53] and osteoarthritis [54]. The effect of the aforementioned modifiers needs to the considered to deliver effective regenerative treatment. With the subclassification of diseases based on patients’ characteristics into various phenotypes and endotypes to facilitate their management [55], a similar categorization of the autologous products based on patients’ characteristics is needed to achieve predictable results.

## 5. Conclusions

Individual factors such as age and comorbid conditions, and procedure factors such as aspirate volume, significantly affect the yield of progenitor cells in BMAC. The sex of an individual patient does not influence the yield of progenitor cells in BMAC. 

## Figures and Tables

**Table 1:** Characteristics of the patients included in the analysis (n = 58).

Variables	Median (IQR)
Age	60 (40–66) years
**Sex**	
Males	31
Females	27
**Comorbidities**	
Diabetes mellitus	9
Rheumatoid arthritis	4
No comorbid illness	45
**Diagnosis**	
Osteoarthritis	32
Diabetic foot ulcers	8
Rheumatoid arthritis	4
Others	14
**BMA Characteritics**	
Aspiration volume	70 (50–80) mL
MNC count	16.27 (11.17–20.66) × 10^6^ cells/10 mL aspirate
CFU count	7.33 (5–12.33) colonies/10 mL aspirate

**Table 2:** Effect of comorbidity on the MNC count of the included patients.

S. No.	Comorbidity	MNC Count (×10^6^ Cells)		X^2^ (df), *p* Value	OR (CI)
		<10	≥10		
1	Present	6 (60)	7 (13.2)	11.247 (1), 0.001	9.85 (2.21 to 43.92)
2	Absent	4 (40)	41 (86.8)		

**Table 3:** Effect of comorbidity on the CFU count of the included patients.

S.No.	Comorbidity	CFU		X^2^ (df), *p* Value	OR (CI)
		<3	≥3		
1	Present	3 (75)	10 (16.9)	7.708 (1), 0.005	14.70 (1.38 to 156.18)
2	Absent	1 (25)	44 (83.1)		

**Table 4:** Correlation heat map of the risk factors analyzed to the yield of BMAC. The heatmap is made of colored cells from red to green to indicate the direction of correlation where red denotes negative correlation and green denotes positive correlation. The intensity of the colors denoted the strength of correlation.

	Age	Sex	Aspiration Volume	Comorbidity	MNC	CFU
**Age**	1					
**Sex**	0.0728	1				
**Aspiration Volume**	0.4243	0.0825	1			
**Comorbidity**	0.1922	0.1519	0.4877	1		
**MNC**	−0.6819	−0.1634	−0.7404	−0.2872	1	
**CFU**	−0.6938	−0.1352	−0.6299	−0.2992	0.95	1

## Data Availability

The data are contained within the manuscript.
